# Determinants and Consequences of Failure of Linkage to Antiretroviral Therapy at Primary Care Level in Blantyre, Malawi: A Prospective Cohort Study

**DOI:** 10.1371/journal.pone.0044794

**Published:** 2012-09-11

**Authors:** Peter MacPherson, Elizabeth L. Corbett, Simon D. Makombe, Joep J. van Oosterhout, Eddie Manda, Augustine T. Choko, Deus Thindwa, S. Bertel Squire, Gillian H. Mann, David G. Lalloo

**Affiliations:** 1 Clinical Group, Liverpool School of Tropical Medicine, Liverpool, Merseyside, United Kingdom; 2 HIV and TB Group, Malawi-Liverpool-Wellcome Trust Clinical Research Programme, Blantyre, Malawi; 3 Faculty of Infectious and Tropical Diseases, London School of Hygiene and Tropical Medicine, London, United Kingdom; 4 HIV Unit, Ministry of Health, Lilongwe, Malawi; 5 College of Medicine, University of Malawi, Blantyre, Malawi; 6 Blantyre District Health Office, Blantyre, Malawi; Boston University, United States of America

## Abstract

**Background:**

Poor rates of linkage from HIV diagnosis to ART initiation are a major barrier to universal coverage of ART in sub-Saharan Africa, with reasons for failure poorly understood. In the first study of this kind at primary care level, we investigated the pathway to care in the Malawian National Programme, one of the strongest in Africa.

**Methods and Findings:**

A prospective cohort study was undertaken at two primary care clinics in Blantyre, Malawi. Newly diagnosed HIV-positive adults (>15 years) were followed for 6-months to assess completion of eligibility assessments, initiation of ART and death. Two hundred and eighty participants were followed for 82.6 patient-years. ART eligibility assessments were problematic: only 134 (47.9%) received same day WHO staging and 121 (53.2%) completed assessments by 6-months. Completion of CD4 measurement (stage 1/2 only) was 81/153 (52.9%). By 6-months, 87/280 (31.1%) had initiated ART with higher uptake in participants who were ART eligible (68/91, 74.7%), and among participants who received same-day staging (52/134 [38.8%] vs. 35/146 [24.0%] p = 0.007). Non-completion of ART eligibility assessments (adjusted hazard ratio: 0.11, 95% CI: 0.06–0.21) was associated with failure to initiate ART. Retention in pre-ART care for non-ART initiators was low (55/193 [28.5%]). Of the 15 (5.4%) deaths, 11 (73.3%) occurred after ART initiation.

**Conclusions:**

Although uptake of ART was high and prompt for patients with known eligibility, there was frequent failure to complete eligibility assessment and poor retention in pre-ART care. HIV care programmes should urgently evaluate the way patients are linked to ART. In particular, there is a critical need for simplified, same-day ART eligibility assessments, reduced requirements for hospital visits, and active defaulter follow-up.

## Introduction

Malawi, with a population of nearly 14 million people is the 10^th^ poorest country in the world and is among the countries hardest hit by the HIV pandemic [1]. The most recent estimates show that 12% of adults are infected with HIV [2]. Despite this bleak picture, Malawi has had tremendous success in scaling-up antiretroviral therapy (ART) delivery using a public health approach that has incorporated decentralisation of treatment delivery to primary health care level, task-shifting from clinicians to nurses, and a nationwide quarterly reporting system [3], [4].

Nevertheless, potential limitations remain. Linkage into HIV care following a positive test is known to be problematic in many African countries [5], and is not tracked in the Malawian programme. Between January 2008 and March 2011, 5,663,852 Malawians received HIV testing and counselling (HTC), with 653,004 (12%) testing HIV-positive [6] and 218,335 (33% of all tested positive) initiated ART [6]. However, the number of those testing positive who were eligible for ART initiation is not captured by the programme, and nor are any data collected on the interval between testing positive and ART initiation, if eligible. Therefore, these very encouraging figures may nonetheless include a substantial proportion of HIV-positive Malawians with failed or unduly delayed ART initiation [7].

Recent studies from Southern Africa have highlighted large gaps in understanding of patient flow in the pre-ART period (between HIV diagnosis and initiation of ART). A systematic review highlighted high rates of attrition before ART initiation [5] which contribute to the high pre- and early-ART mortality rates observed in many sub-Saharan African programmes [8], [9]. Only two prospective cohort studies have examined linkage to ART in sub-Saharan Africa [10], [11], both based in South Africa. No studies to date have described outcomes for individuals tested in primary care, where HIV services are increasingly delivered.

This prospective cohort study was undertaken in primary health care clinics in Blantyre Malawi. The objectives were to describe progression throughout the HIV care pathway under programmatic conditions, to estimate the proportion of patients diagnosed HIV-positive who completed ART assessments and started ART. We also aimed to identify key points of attrition from the HIV care pathway and to investigate individual risk factors for failure.

## Methods

### Ethics Statement

Ethical approval for the study was obtained from the Research Ethics Committees of the College of Medicine of Malawi and Liverpool School of Tropical Medicine. All participants provided written informed consent (or independently-witnessed thumbprint for illiterate participants). Both Research Ethics Committees approved witnessed thumbprint consent for illiterate participants. We did not require written (or witnessed thumbprint) informed consent from the guardian of participants aged between 16 and 18 years old. In the Malawian national HIV care guidelines, guardian consent is not required for HIV testing for individuals aged 15 years and over. Both Research Ethics Committees approved this consent procedure for participants between 16 and 18 years old.

### Study Design, Population and Procedures

A prospective cohort study was carried out between January and September 2011. Two primary health care clinics (Ndirande Health Centre and Chilomoni Health Centre) located in the northwest of Blantyre were selected as study sites as they were the largest primary clinic providers of ART, having initiated a cumulative total of 5720 people onto ART [6].

During the 3-month recruitment period, study research assistants undertook exit interviews with clinic attendees immediately after HTC. Interviews were held in a private room. Individuals who met study eligibility criteria (>15 years old with confirmed HIV infection) were invited to participate. Due to clinic workload and limited availability of private space for interview, a maximum of 6 participants were recruited from each clinic per day.

Trained research assistants administered a baseline questionnaire, extracted clinical data from patient-carried records and clinic HTC registers and carried out WHO clinical staging. Participants were then advised to follow instructions for further HIV care given to them by routine clinic staff. To obtain an estimate of total clinic attendances during the study period, records of all adult (>15 years) clinic attendances were extracted from clinic registers.

In order to minimise risk of inadvertent disclosure of HIV status during follow-up, participants were asked to consent to follow-up by home-based tracing in the event of non-return by the end of the follow-up period. All participants consented to home-based tracing.

### Ascertaining Outcomes on the HIV Care Pathway

At the time of the study, Malawian ART eligibility criteria were based on WHO clinical staging (eligible if stage 3 or 4), with CD4 criteria of <250 cells/ul if in WHO stage 1 or 2 [12]. Guidelines were the same for pregnant women, except that single dose nevirapine and one week of zidovudine were given if not started on triple therapy. In the clinics, HTC is performed by counsellors and, following HIV diagnosis, patients are referred to clinicians (clinical officers, nurses and midwifes) for WHO staging. Patients in WHO stage 1/2 were referred for CD4 count measurement by the assessing clinician. At the time of the study, completion of CD4 count measurement required at least two attendances at the city’s central hospital: one to have blood drawn and one to collect the result. The patient then brought the CD4 count result back to the primary health care centre for clinician review. Following ART initiation, patients attended routine follow-up on a monthly basis at the clinics.

Guidelines for pre-ART care recommended life-long follow-up through general medical outpatient clinics with daily co-trimoxazole prophylaxis and 6-monthly reassessment for ART eligibility. In this study, “retained in pre-ART care” was defined as: currently taking co-trimoxazole or having collected co-trimoxazole in the last 3-months; or having attended an ART support group meeting or pre-ART education classes in past 3-months; and not having died or been lost to follow-up. Participants who “transferred” to another clinic, but who were traced to home were defined as retained in care. Participants who did not attend either study clinic for 3- and 6-month appointments, and who could not be traced after three home visits at both 3 and 6 months, were defined as lost to follow-up.

Participants were followed-up at 3-months and 6-months after their HIV diagnosis with questionnaires and inspection of clinic registers and patient carried records. For participants who had not returned to the clinic by the end of each follow-up period, research assistants commenced telephone and home tracing. At home visits, questionnaires and review of patient carried records were carried out. Where the participant had died, household members were asked to provide the date of death and the participant’s patient-carried record.

### Statistical Methods

Self-rated general health was assessed on a 4-point Likert-type scale [13]. A household wealth scale was constructed using the proxy means test method [14], based on the 1998 Malawi Integrated Household Survey [15]. The proxy means test approach uses eight household variables that have been shown to be correlated with the consumption level of the household, thus allowing participants to be wealth-ranked on their ability to meet household consumption needs [16].

Participant characteristics were stratified into two groups (pregnant women vs. men and non-pregnant women) as the clinic pathway for these groups were different (pregnant women received provider-initiated HTC in the antenatal clinic whereas men and non-pregnant women were tested in the outpatient clinics) and baseline characteristics of men and non-pregnant women were found to be similar.

Time-at-risk in the study was estimated as the period between date of HIV diagnosis and date of ART initiation, or completion of 6-months follow-up or death in the event of non-initiation of ART. Data on participants who were lost-to-follow-up were right-censored at the midpoint between their last follow-up assessment and the date of their next expected follow-up visit. Cox regression analysis was used to examine associations with initiation of ART. In constructing the multivariate regression model, variables significant at the p<0.10 level were included using a forward step-wise procedure.

## Results

### Clinic Attendances, HTC Episodes and Cohort Characteristics

**Figure 1:**
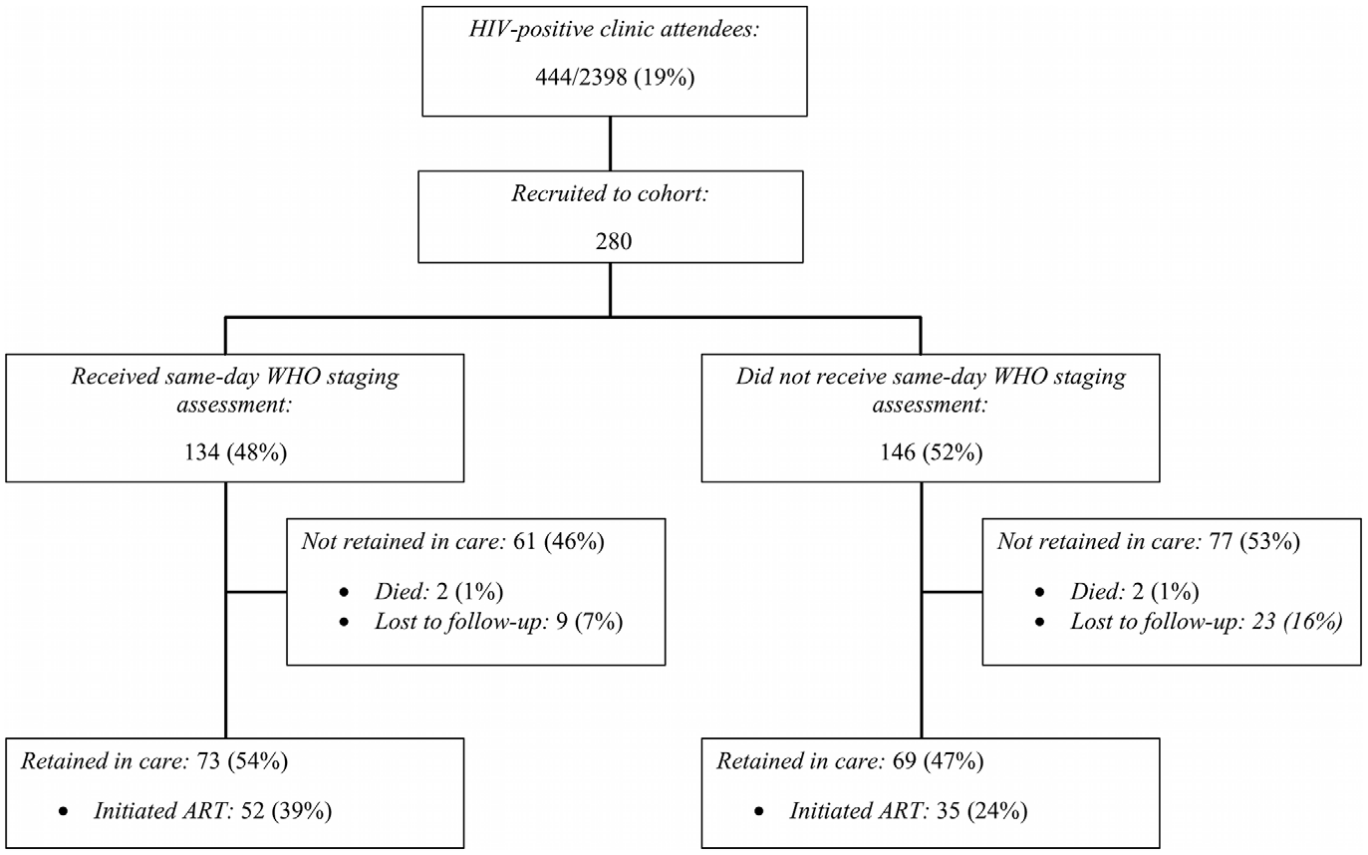
Flow diagram of cohort outcomes. ART: antiretroviral therapy.

During the three months recruitment period (January to April 2011), there were 18,021 adult (>15 years old) attendances at the two study primary care clinics Baseline characteristics have been previously reported [17]. In brief, there were 2,398 HTC episodes during the 18,021 clinic attendances (13.3%) and 444 confirmed HIV infections, giving a positivity rate of 18.5% (Figure 1).

Following completion of HTC procedures, 280 newly diagnosed HIV-positive adults were recruited in to the cohort, with 84 declining to participate and 80 not recruited because of exceeding the study recruitment limit of six participants per day (Figure 1). Seventy-five per cent of participants were women (211/280), of whom 57.3% (121/211) were pregnant (Table 1). Pregnant women were more likely to be younger (median 25 years [IQR: 21–30] vs. 31years [interquartile range [IQR]: 27–37]) unemployed (71.1% vs. 44.7%), to have previously tested for HIV (57.9% vs. 37.1%), to be married (90.9% vs. 66.7%) and to be from a relatively wealthier household (27.7% vs. 23.7% in wealthiest quartile) than men and non-pregnant women. On self-rated general health (18.9% vs. 1.7% self-rated poor), study assessed WHO clinical stage (81.1% vs. 37.2% in WHO stage 3 or 4) and CD4 count (median 240 cells/ul [IQR: 143–376] vs. 373 cells/ul [236–527]), men and non-pregnant women received HTC at a more advanced stage of illness than pregnant women (Table 1).

**Table 1:** Baseline characteristics of cohort members.

Characteristic	Men and non-pregnant women	%	Pregnant women	%
*Total*	159	100	121	100
*Age (years) – median (IQR)*	31	27–37	25	21–30
*Marital status*				
Married	106	66.7	110	90.9
Divorced	24	15.1	2	1.7
Never married	18	11.3	8	6.6
Widowed	11	6.9	1	0.8
*BMI (kg/m^2^) – median (IQR)*	21	19–23	24	22–26
*Employment status*				
Not in formal employment	71	44.7	86	71.1
In formal employment	88	55.3	35	28.9
*Literacy*				
Able to read a newspaper	129	81.1	98	81.0
Illiterate	30	18.9	23	19.0
*Wealth quartile* ±				
Poorest quartile	42	31.1	14	14.9
Next poorest quartile	35	25.9	23	24.5
Next wealthiest quartile	26	19.3	31	33
Wealthiest quartile	32	23.7	26	27.7
*Previously tested for HIV*				
Yes	59	37.1	70	57.9
No	100	62.9	51	42.1
*Tuberculosis history*				
Current	6	3.8	0	0
Previous	9	5.7	6	5.0
Never	144	90.6	115	95.0
*Self-rated general health*				
Excellent	21	13.2	48	39.7
Good	38	23.9	51	42.1
Fair	70	44	20	16.5
Poor	30	18.9	2	1.7
*WHO clinical stage* *				
Stage 1 or 2	30	18.9	76	62.8
Stage 3 or 4	129	81.1	45	37.2
*Received same day WHO staging*	89	56.0	45	37.2
*ART eligibility at 6-months* ♯				
ART eligible	136	85.5	47	38.8
Not ART eligible	7	4.4	12	9.9
Not fully assessed	16	10.1	62	51.2
*CD4 count (median, IQR) cells/ul* §	240	143–376	373	236–527

± Proxy means test for household wealth is a continuous variable constructed from: asset ownership (fridge, car or motorbike, bed); mean household size; squared mean household size; mean age of household head; education level of household head; mean number of salaried household members; household lighting source in electricity or gas. Categorised into quartiles to describe differences in wealth between groups.

* Study assessed WHO stage: participants were independently staged by research assistants.

♯ ART eligibility defined as meeting national ART criteria (CD4<250 cells/ul or WHO stage 3 or 4) on routine clinic assessments.

§ Where blood taken and results available at primary clinic (men and non-pregnant women [n = 51], pregnant women [n = 30]).

### Primary Cohort Outcome: ART Initiation at 6-Months

**Figure 2:**
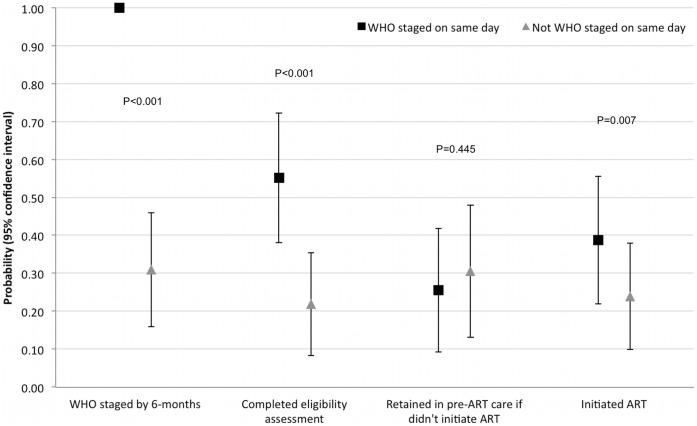
Completion of pre-ART care stages if received same-day WHO clinical staging.

The 280 cohort participants were followed-up for 82.6 patient-years. By 6-months, 87/280 (31.1%) had initiated ART, with 32 (11.4%) lost to follow-up (Figure 1). There were 25/89 (28.1%) initiations from participants who were assessed as WHO stage 1 or 2 by same-day WHO staging, and 27/45 (60.0%) of those who were WHO stage 3 or 4. Overall participants who did not receive same-day WHO staging assessment were substantially less likely to initiate ART (35/146 [24.0%] vs. 52/134 [38.8%], p = 0.007– Figure 2). Twenty five per cent (6/24) participants who were assessed by clinicians as not ART eligible initiated ART.

Of the 91 participants who had met national ART eligibility criteria through routine clinic assessments over 6-months, 68 (74.7%) initiated ART. The median number of days to ART initiation was 47 (interquartile range [IQR]: 21–74). Men (34 days, IQR: 19–57) had a shorter time to ART initiation than pregnant (median 56 days, IQR: 47–87), and non-pregnant women (median 49 days, IQR: 21–85). This may have been because pregnant women were more likely to be assessed in WHO stage 1 or 2 and to have a higher median CD4 count (Table 1).

**Figure 3:**
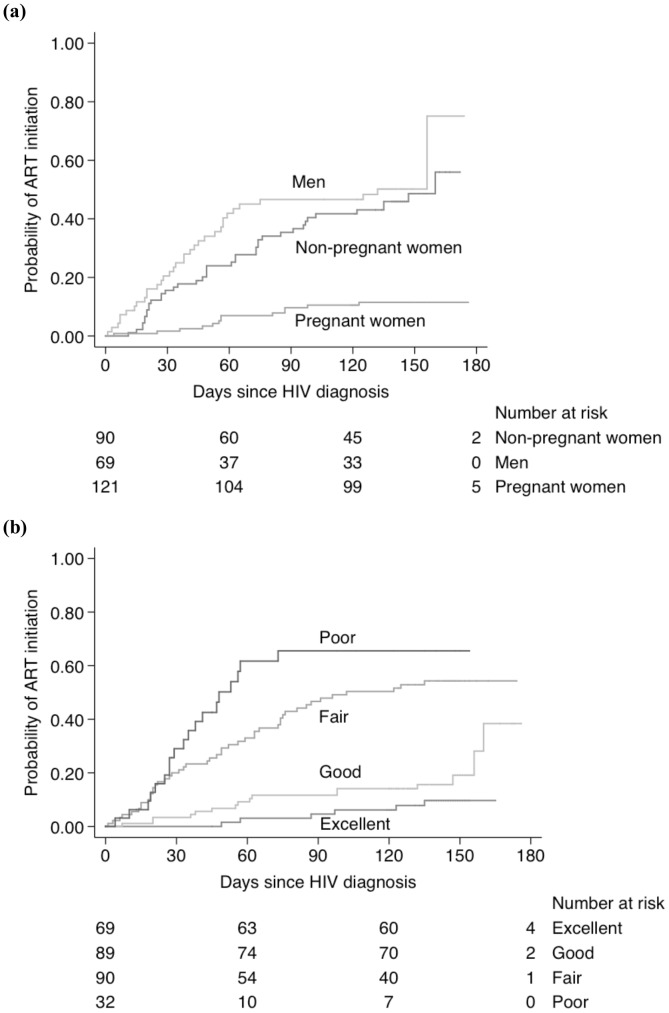
Kaplan-Meier plots of initiation of ART by (a) sex and pregnancy; and (b) self-rated general health.

Kaplan-Meier plots (Figure 3) showed that ART initiation was higher in men (210 per 100 person-years, 95% CI: 122–226) and non-pregnant women (166 per 100 person-years, 95% CI: 150–294) compared to pregnant women (31 per 100 person-years, 95% CI: 18–53; logrank test: p<0.001). Participants who had poor (350 per 100 person-years, 95% CI: 223–548) or fair (212 per 100 person-years, 95% CI: 159–283) self-rated general health had uptake of ART compared to participants who had self-rated good (53 per 100 person-years, 95% CI: 32–87) or excellent (24 per 100 person-years, 95% CI: 11–53) general health (logrank test: p<0.001).

### Factors Associated with ART Initiation

On univariate analysis (Table 2), men (hazard ratio [HR]: 1.29, 95% CI: 0.81–2.04), older participants (HR: 1.05, 95% CI: 1.04–1.07), participants who had fair (HR: 8.48, 95% CI: 3.62–19.88) or poor (HR: 13.70, 95% CI: 5.44–34.51) self-rated general health, participants in WHO stage 3 or 4 (HR: 3.25, 95% CI: 1.89–5.60) and participants who received same-day WHO staging (HR: 1.66, 95% CI: 1.08–2.55) were more likely to initiate ART. Pregnant women (HR: 0.20, 95% CI: 0.11–0.40), participants who were assessed as not ART eligible (HR: 0.16, 95% CI: 0.07–0.36) and who didn’t complete ART eligibility assessments (HR: 0.07, 95% CI: 0.04–0.12) were less likely to initiate ART.

**Table 2:** Univariate and multivariate associations with ART initiation over 6-months (n = 280).

	Univariate hazard ratio	95% CI	P-value	Multivariate hazard ratio	95% CI	P-value
*Sex and pregnancy*						
Female non-pregnant	1					
Male	1.29	0.81–2.04		1.29	0.78–2.14	
Female pregnant	0.20	0.11–0.40	<0.001	0.58	0.28–1.21	0.316
*BMI (kg/m^2^) – continuous*	0.83	0.77–0.89	<0.001	0.96	0.89–1.04	0.312
*Age (years) - continuous*	1.05	1.04–1.07	<0.001	1.01	0.99–1.04	0.341
*Employment status*						
Not in formal employment	1					
In formal employment	1.25	0.81–1.90	0.306			
*Literacy*						
Able to read a newspaper	1					
Illiterate	0.81	0.46–1.43	0.459			
*Wealth quartile* *						
Poorest quartile	1					
Next poorest quartile	1.01	0.55–1.83				
Next wealthiest quartile	0.75	0.39–1.43				
Wealthiest quartile	0.61	0.31–1.20	0.372			
*Self-reported general health*						
Excellent	1			1		
Good	2.21	0.86–5.64		1.70	0.65–4.49	
Fair	8.48	3.62–19.88		2.87	1.11–7.40	
Poor	13.70	5.44–34.51	<0.001	3.16	1.11–8.93	0.104
*Study assessed WHO clinical stage*						
Stage 1 or 2	1			1		
Stage 3 or 4	3.25	1.89–5.60	<0.001	1.00	0.54–1.85	0.992
*Health facility*						
Chilomoni Health Centre	1			1		
Ndirande Health Centre	0.66	0.42–1.02	0.056	0.94	0.46–1.95	0.876
*Received same-day WHO staging*	1.66	1.08–2.55	0.019	0.72	0.36–1.43	0.349
*Eligibility assessment at 6 months*						
* ART eligible*	1			1		
* Not ART eligible*	0.16	0.07–0.36		0.19	0.08–0.44	
* Not fully assessed*	0.07	0.04–0.12	<0.001	0.11	0.06–0.21	<0.001

* Proxy means test for household wealth: variable constructed from asset ownership (fridge, car or motorbike, bed), mean household size, squared mean household size, mean age of household head, education level of household head, mean number of salaried household members, household lighting source is electricity or gas. Constructed as a continuous measure with higher values representative of wealthier household.

On multivariate adjusted analysis failure to complete ART eligibility assessments (adjusted HR: 0.11, 95% CI: 0.06–0.21) or assessment as not ART eligible (adjusted HR: 0.16, 95% CI: 0.07–0.36) were significantly associated with non-initiation of ART. Fair (adjusted HR: 2.87, 95% CI:1.11–7.40) or poor self-rated general health (adjusted HR: 3.16, 95% CI: 1.11–8.93) were associated with ART initiation. However, male sex (adjusted HR: 1.29, 95% 0.78–2.14), pregnancy in women (adjusted HR: 0.58, 95% CI: 0.28–1.21), and age (adjusted HR: 1.01, 95% CI: 0.99–1.04) were no longer associated with ART initiation.

### Evaluation of Patient Flow Towards ART Initiation and Retention in Pre-ART Care for Non-ART Eligible Participants

Following HIV diagnosis, routine clinic WHO clinical staging was completed on the same day for 134 (47.9%) participants with the remainder asked to return to the clinic on an alternative day when a clinician was available, or reporting no clear instructions. By 6-months, a further 45 (16.1%) had completed routine clinic WHO staging. Overall, 61/179 (34.1%) were in WHO stage 3 or 4.

In total, 153/280 (54.6%) participants were referred by health workers for CD4 counts measurement, of whom 99 (64.7%) had blood taken and 81 (52.9%) had received results by 6-months. The median CD4 count was 294 cells/ul (interquartile range: 173–473 cells/ul) and 40/57 (70.2%) had a CD4 count of <250 cells/ul. Median CD4 count was lower in men and non-pregnant women (240 cells/ul, IQR: 143–376) than in pregnant women (373 cells/ul, IQR: 236–527, p = 0.039). The median delay from HIV diagnosis to ART initiation for participants with CD4 count <250 cells/ul was 63 days (IQR: 47–81 days).

Overall, 91 (32.5%) participants had met National ART criteria by 6-months, with 30 (10.7%) not ART eligible and 159 (56.8%) not fully assessed. Failure to complete assessments was higher in pregnant women (95/121, 78.5%) compared to men (27/69, 39.1%) and non-pregnant women (37/90, 41.1%, p<0.001).

Participants who were not WHO staged on the same day as HIV diagnosis were significantly less likely to complete ART eligibility assessments than participants who had same-day WHO staging (74/134 [55.2%] vs. 32/146 [21.9%]; p<0.001– Figure 3). By 6-months, participants who received same-day WHO staging were more likely (although not statistically significant) to be retained in any HIV care (either initiated ART or retained in pre-ART) than participants who did not receive day WHO staging (71/134 [54.5%] vs. 69/146 [47.3%]; p = 0.288).

Retention in pre-HIV care was poor for the 193 participants who had not initiated ART by 6-months: 138 (72%) had dropped out, with 4 (2.1%) deaths. Drop-out from pre-ART care did not vary significantly by gender or pregnancy (data not shown). Of the 55 participants retained in pre-ART care, 28 (50.9%) had collected co-trimoxazole within the last 3-months.

### Mortality during Cohort Follow-up

By 6-months, 15/280 (5.4%) participants had died (mortality rate 14.8 per 100 person-years, 95% CI: 8.9–24.6 per 100 person-years). Eleven of fifteen deaths (73.3%) occurred following ART initiation. The median time from HIV diagnosis to death in participants who initiated ART was 40 days (IQR: 39–48) and from ART initiation to death was 38 days (IQR: 24–103 days).

## Discussion

The main finding from this study, which is the first prospective cohort from primary care level, and the first prospective study outside of South Africa, was that linkage to ART was high among individuals who were promptly assessed for ART eligibility and found to be ART eligible, but that outcomes were substantially worse for individuals who did not complete same-day ART eligibility assessments, with extremely low uptake of ART and poor retention in pre-ART care. Although completion of ART eligibility assessment was suboptimal for all groups, pregnant women fared particularly badly. This emphasises the need to simplify and streamline ART eligibility assessments with an urgent need to prioritise same-day assessment in National programme policies.

The high reported risk of failure of linkage from HIV diagnosis to initiation of ART following a positive HIV test in sub-Saharan Africa is based on a small number of mostly retrospective studies [10], [11], [18], [19], [20], [21], [22], [23], [24], [25], [26], [27], [28] with a systematic review estimating that only one-third to one-sixth of patients will complete all steps in the HIV care pathway and initiate ART [5]. The authors concluded by calling for more prospective data from high HIV prevalence countries in order to inform policy [5].

The lack of capacity for same-day ART eligibility assessments appears to be the key barrier in this setting: only half of participants were WHO clinically staged by 6-months and successful completion of CD4 count measurement was extremely low. Clinical staging was originally intended to be used as a diagnostic tool for HIV in low resource settings prior to the widespread availability of rapid test kits [29], and was then adopted for defining endpoints in clinical trials. The WHO clinical staging system is known to have a relatively low sensitivity for identifying the currently recommended CD4 cut-off for ART initiation [30] and includes a number of conditions that cannot be diagnosed in most resource poor settings. It is therefore unnecessarily time-consuming and overly complex for use as an ART eligibility assessment tool and as our data suggests, this results in frequent failure to stage immediately after testing HIV-positive.

Only one-third of participants referred for CD4 count measurement returned to the clinic with a result. This was worse than reported in a hospital-based HIV cohort in South Africa, where about one-half returned with a result [11]. We attribute the low completion of CD4 measurement reported here to the greater resource limitations in Malawi requiring participants to travel to the city’s central hospital for venepuncture (with the lab machine often out of action) and a further return trip required to collect results. Other studies on HIV/TB integration in Malawi have shown a pronounced effect on retention in care when travel between clinics is required, a process that is known to be expensive and time-consuming [20].

Ideally, all patients should be guaranteed same-day ART eligibility assessment following HIV diagnosis, without the need to visit multiple clinics. Using a “test and treat” approach is one option [31]. Alternatively, we suggest development and validation of clinical algorithms expressly designed for ascertaining ART eligibility status and with sufficient simplicity to be carried out by front-line health workers, including community health workers. If CD4 counts are required, they should be point-of-care [32], [33]. Pre-ART care registers directly completed once HIV infection has been confirmed would allow appropriate monitoring and reporting of linkage and retention, and tracing of patients lost to care [34].

Also notable is our finding that pregnant women were at particularly at risk of failure to complete ART eligibility assessments and were less likely to initiate ART. Health systems factors (especially crowded antenatal clinics, staff shortages and inaccessibility of CD4 count measurements) were reported as being the major barriers to pregnant women’s completion of ART eligibility assessments in a linked qualitative study [17]. Pregnant women underwent HTC at an earlier stage of HIV infection as shown by WHO stage, CD4 count (where completed) and self-rated general health and this may explain why they were less likely to initiate ART than men and non-pregnant women. Longer-term follow-up of pregnant women’s uptake of ART will be required to confirm this. Nevertheless, These findings have major implications for efforts to reduce maternal mortality and eliminate mother-to-child transmission, although in Malawi, following the completion of this study, universal life-long triple therapy for HIV-positive pregnant women (“Option B+”) has been introduced, hopefully bypassing the problems of suboptimal ART eligibility assessments and delayed ART initiation [35].

All of our participants were extremely poor, but greater poverty was not a significant risk factor for failure to initiate ART. There was a trend towards failure to be retained in pre-ART care among our poorest participants. Other studies in Malawi show that TB suspects spend a large proportion of their annual household income simply to complete the investigations required to access TB treatment [36]. Although we did not investigate expenditure here, it is likely that the cost of making repeat clinic visits required for ART eligibility assessment are similarly financially draining and so likely to contribute to drop-out from HIV care-seeking.

Mortality in our cohort was 5%, with most deaths occurring after initiation of ART. There are few other data on mortality during pre-ART care-seeking [26]. The immediate post-ART initiation period is known to be high risk for mortality [8], especially for patients with advanced immunosuppression. Diagnosing HIV early in the course of infection and reducing delays to treatment initiation reduces mortality in observational studies [37].

There were a number of limitations to this study. Only patients attending at two primary health care centres were studied, although these clinics are the two largest primary care providers of ART in Blantyre. Our results may not be generalizable to HIV programmes in other settings that are structured differently. We did not measure CD4 counts in this study, as this would have constituted an intervention in the care-seeking process. As such, we cannot ascertain the true proportion of individuals who were eligible for ART. We were able to recruit a maximum of six participants per clinic per day due to time and space constraints. This may have resulted in some selection bias in participant recruited. However, as previously reported [17], characteristics of HIV participants recruited and not recruited to the study were similar. Reasons for undergoing HTC may have influenced linkage to ART, but sufficient data on testing behaviour was not captured in this study to allow this to be explored. With telephone and home tracing, our loss to follow-up was 11%, which is lower than the majority of other studies investigating pre-ART patients, but may conceal missed events such as initiation of ART or death. Mortality may be an important cause of pre-ART attrition and larger studies are required to give further insights into the extent of and factors associated with death.

In summary, this prospective cohort study recruiting newly diagnosed HIV-positive patients from primary care has identified the critical need for same-day ART eligibility assessments, with need for point of care CD4 counts if these are to be used as part of staging. Failure to provide same-day staging resulted in poor completion of ART assessments and high rates of drop-out from care. HIV care programmes in this region should revaluate the way that testing is linked to care, with concerted effort clearly needed to improve rates of linkage to ART. This should include guarantee of a same-day, simplified ART eligibility assessment (potentially incorporating point of care CD4 measurement) and patient registers that cover the whole HIV care pathway.
